# Molecular Dynamics Reveals a Glycan Conformation Underlying IDUA Dysfunction in Attenuated MPS I

**DOI:** 10.1111/jcmm.71396

**Published:** 2026-10-11

**Authors:** Maria Giovanna Eva Papadopoulos, Giuseppe Bonapace, Lorenzo Taglietti, Barbara La Ferla, Rosanna Rizzi, Michele Pitaro, Daniela Concolino, Domenico Alberga, Giuseppe Felice Mangiatordi

**Affiliations:** ^1^ CNR–Institute of Crystallography Bari Italy; ^2^ Department of Health Sciences University Magna Graecia Catanzaro Italy; ^3^ Department of Biotechnology and Biosciences Milano‐Bicocca University Milan Italy; ^4^ Department of Earth and Environmental Sciences Milano‐Bicocca University Milan Italy; ^5^ Istituto Nazionale Biostrutture e Biosistemi (INBB) Rome Italy

## Abstract

Mucopolysaccharidosis Type I (MPS I) is a rare autosomal recessive lysosomal storage disorder caused by mutations in α‐ʟ‐iduronidase (IDUA) enzyme, a glycosylated hydrolase responsible for glycosaminoglycan (GAG) degradation. Pathogenic variants are associated with a broad clinical spectrum, from severe Hurler syndrome to attenuated Scheie phenotype. The missense mutations L490P and P533R cause attenuated‐intermediate MPS I, retaining residual enzyme activity. To investigate the structural mechanisms underlying these phenotypes, we performed all‐atom molecular dynamics simulations –18 trajectories of 1.6 μs each– comparing wild type IDUA with L490P and P533R mutants at pH 7.5 and 4.5, mimicking the endoplasmic reticulum (ER) and lysosomal microenvironments, respectively. Backbone root‐mean‐square deviation and fluctuation analyses indicated that the overall protein fold was maintained across conditions; however, we identified a pH‐dependent restricted conformational flexibility of the N372‐linked glycan in both mutants. Cluster analysis performed on the obtained trajectories showed that both mutations affect the conformation of this glycan. Hydrogen bond analysis further revealed that P533R induces aberrant, persistent glycan–protein interactions, providing a possible structural explanation for reduced IDUA activity and, therefore, GAG processing. Our findings, supported by in vitro data, propose glycan conformational restriction as a possible new molecular mechanism that underpins enzymatic dysfunction in attenuated MPS I, opening new avenues for structure‐informed therapeutic strategies.

**Abbreviations**
GAG: glycosaminoglycan
IDUA: α‐ʟ‐iduronidase
LSD: lysosomal storage disorder
MD: molecular dynamics
MPS I: mucopolysaccharidosis type I
WT: wild type

## 1 Introduction

Mucopolysaccharidosis Type I (MPS I) is a rare autosomal recessive lysosomal storage disorder caused by mutations affecting α‐ʟ‐iduronidase (IDUA), a lysosomal enzyme encoded by the human α‐ʟ‐iduronidase gene (*hIDUA*) [1, 2]. The enzyme belongs to glycosyl hydrolase family 39 [3]. IDUA is synthesized at the endoplasmic reticulum (ER), traverses the Golgi, where it undergoes *N*‐glycosylation and proteolytic processing, and is trafficked to the lysosomes (Figure 1) [5, 6]. It is responsible for the hydrolysis and degradation of the α‐linked ʟ‐iduronic acid (IdoA) residues from glycosaminoglycans (GAGs), including dermatan sulphate and heparan sulphate [3, 7, 8]. This enzyme deficiency leads to the accumulation of GAGs within lysosomes, resulting in impaired cellular function and multisystem damage, with clinical severity ranging from mild to severe [9].

**FIGURE 1:**
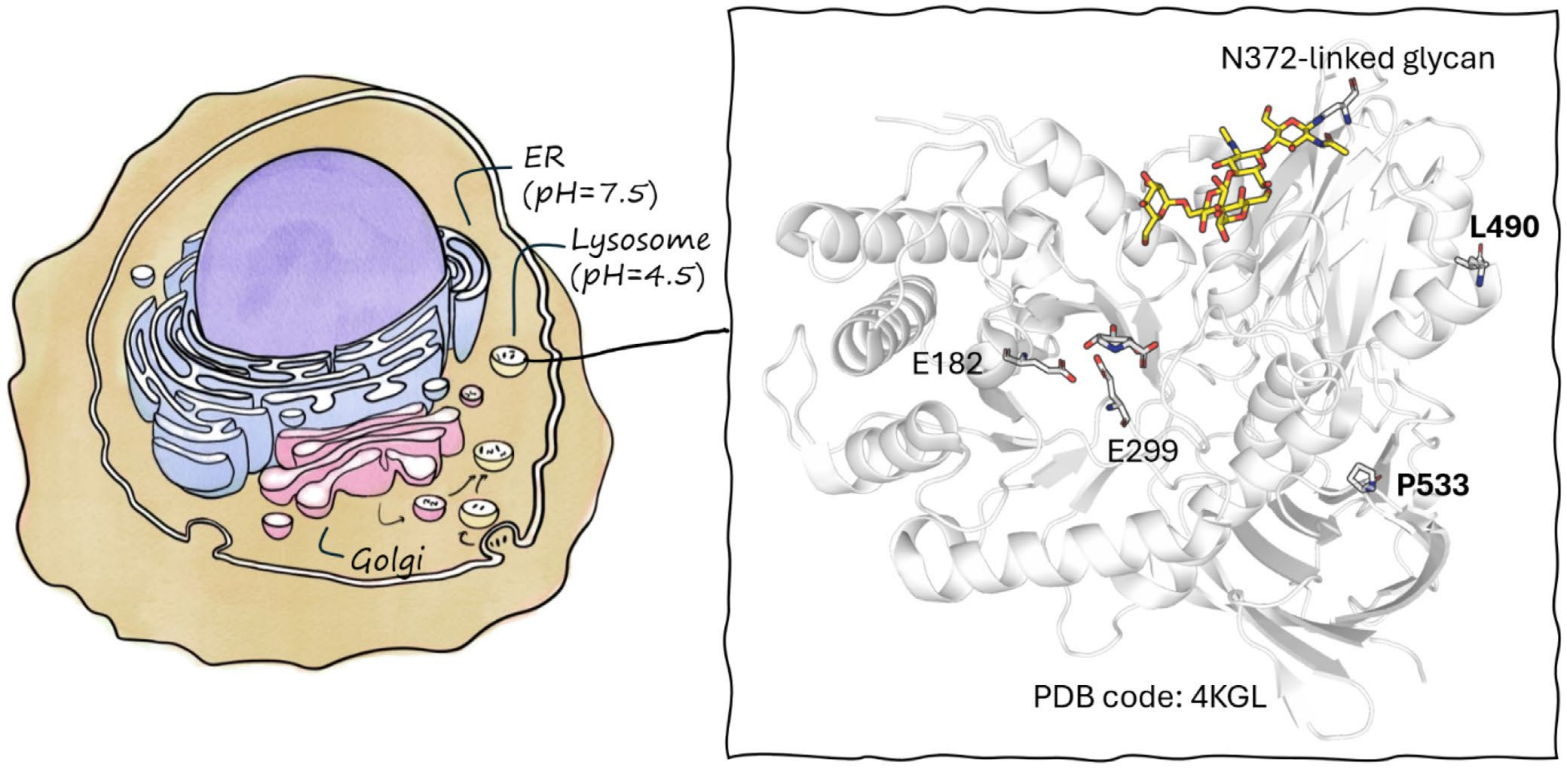
Left: Illustration of a cell and environmental pH change of IDUA from the ER (pH = 7.5) to the Golgi apparatus and lysosome (pH = 4.5), where it exerts its function. Right: Three‐dimensional structure of IDUA (PDB code: 4KGL [4]); the key catalytic residues, E182 and E299, mutated residues under study, L490 and P533 and ligand IdoADNJ in sticks, while N372‐linked glycan shown in yellow sticks.

MPS I is classified into three phenotypic forms: Hurler syndrome (severe) [10], Hurler–Scheie syndrome (intermediate), and Scheie syndrome (attenuated) [11, 12]. Severe forms are characterized by early‐onset neurodegeneration, skeletal abnormalities and reduced life expectancy, whereas attenuated forms present later in life with milder manifestations such as joint stiffness, corneal clouding, and cardiac involvement, allowing for a longer life expectancy [2].

The three‐dimensional structure of IDUA was first resolved by Maita et al. in 2013, in both the *apo* form and in complex with α‐ʟ‐iduronic acid, using X‐ray crystallography [13]. In the same year, Bie et al. elucidated the mechanism of action of the enzyme via X‐ray crystallography, using a covalently bound ligand at the active site [4]. The enzyme is composed of a triose‐phosphate isomerase (TIM) barrel domain (residues R48‐A394), where the active site resides, with key residues E182 and E299, a β‐sandwich domain (E398‐A542), and a Type III fibronectin‐like domain (T555‐E640) [4]. Lastly, six glycosylation sites were identified, namely N110, N190, N336, N372, N415 and N451. Among the six glycosylation sites, N372 is functionally unique; it is composed of five saccharides, specifically three mannose (MAN) and two N‐acetylglucosamine (NAG) residues (MAN_3_NAG_2_), located on the TIM barrel, and interacts with the substrate [13]. *N*‐glycosylation is widely recognized as one of the most prevalent and functionally important post‐translational modifications, contributing to protein folding, stability, intracellular trafficking and diverse cellular functions [14, 15].

Among pathogenic IDUA variants, L490P and P533R [2, 16, 17, 18, 19, 20] (located at the edge of α15 helix and at a loop of the strands β23 and β24 of the β‐sandwich domain, respectively), are commonly associated with attenuated and intermediate phenotypes (Hurler–Scheie and Scheie) [21]. P533R may lead to local backbone destabilization of the β‐sandwich domain and the neighbouring 15α‐helix, due to the introduction of the bulkier and positively charged arginine opposed to the proline [4, 22]. Instead, L490P could lead to both disruption of local hydrophobic contacts that arose from leucine, as well as truncation or helix kink or rotation due to the insertion of the proline [23]. These mutations retain partial IDUA activity, thereby reducing systemic disease severity; however, they can still lead to mild neurological impairments, including cognitive delay and attention deficits, due to GAG accumulation in the central nervous system (CNS) [7].

Current treatments, including enzyme replacement therapy (ERT) [24, 25, 26] and haematopoietic stem cell transplantation (HSCT) [27, 28], can ameliorate somatic symptoms but are less effective in addressing central nervous system (CNS) involvement due to the limited ability of enzymes to cross the blood–brain barrier (BBB) [29]. Emerging therapeutic strategies, such as prenatal ERT, or in utero enzyme replacement therapy (IUERT) [25], and gene therapy (GT) [30, 31], such as OTL‐203 [32], are promising; however, some gene therapy approaches, such as RGX‐111 [33], are still in early development or have shown limited clinical efficacy, and remain costly and require further validation for MPS I. A major challenge in the development of effective therapies lies in the still incomplete understanding of the molecular mechanisms underlying disease onset and progression, particularly those associated with IDUA dysfunction. The lack of detailed insight into how specific mutations impair enzyme structure and function hampers the rational design of targeted and durable therapeutic strategies. Previous in silico studies employing various computational approaches, including molecular dynamics (MD) simulations, provided the first insights into the molecular basis of IDUA enzymatic dysfunction. However, these investigations were limited by relatively short simulation times and did not account for the distinct pH environments encountered by IDUA during its maturation and trafficking from the ER to the lysosome [22, 34, 35, 36, 37].

To address this gap, in this study, we aimed to better understand the molecular mechanisms and conformational changes that underlie the loss of enzymatic activity, focusing in particular on the L490P and P533R mutants. We explored putative structural ‘switches’ driving loss of enzymatic function in the mutated IDUA using MD simulations. We compared the wild type (WT) apo protein to the aforementioned mutants to uncover potential micro‐switches that may promote protein misfolding and/or induce conformational changes resulting in impaired enzyme function. Here, we investigated the structural mechanisms underlying mutation‐induced IDUA dysfunction under different pH conditions and identified the conformation of the N372‐linked glycan as a key molecular determinant of enzymatic impairment. Preliminary in vitro measurements of residual enzyme activity further support the predicted mutation‐dependent behaviour.

## 2 Methods

### 2.1 Model System Preparation

The experimentally solved structure via X‐ray of IDUA in complex with [2R,3R,4R,5S]‐2‐carboxy‐3,4,5‐trihydroxy‐piperidine (IdoADNJ; PDB CODE: 4KGL [4]) was used to prepare both WT and mutants.

The single‐point mutations, namely L490P and P533R, were obtained using the ‘Mutate Residue’ from Maestro [38], Schrödinger Suite. The structure was stripped of water molecules, crystallization additives and IdoADNJ; the protein, chain B, along with the glycans, chains E and F, were prepared using the ‘Protein Preparation Workflow’ [39, 40] and hydrogen atoms were assigned at both pH 7.5 (±2.0) and 4.5 (±2.0) using default settings. Any missing side chains were completed and the most favourable protonation state for each amino acid was assigned. The complexes were finally minimized using the S‐OPLS (OPLS4) force field [41].

### 2.2 Molecular Dynamics Simulations

The systems were initially prepared as described in ‘System preparation,’ in Maestro. The systems, specifically WT, L490P and P533R, were prepared at pH 7.5 and 4.5 (Table S1). Afterwards, the systems were inserted into a water box (TIP3P) with a volume of ~10^3^ Å^3^ around the protein, incorporating the protein and neutralizing it with 0.15 M NaCl, using ‘System Builder’ and an initial minimization step was performed using ‘Minimization’ for 100 ps via Desmond v2.3 in Schrödinger Suite, 2024‐4 [42, 43]. The simulations were executed on the HERMES computer cluster [44] via Desmond, with approximately 97,500 atoms. The systems were minimized and relaxed ahead of the production step, under NPT conditions at 1 atm until a temperature of 310 K was reached. A Nosé‐Hoover thermostat and Martyna‐Tobias‐Klein barostat with relaxation times of 1 ps and 2 ps were used, respectively. For the Coulombic interaction, a cut‐off of 9.0 Å was used.

After preparing the complete systems, the protonation states at pH = 7.5 were as follows: (i) histidines: neutral Nδ for residues H30, H82, H91, H113, H171, H186, H242, H356, H358, H378, H416, H572, neutral Nε for H139, H226, H240, H425, H449, H539 and protonated for H58, H185, H262, H329; (ii) aspartates: D35, D62, D69, D115, D119, D142, D145, D154, D184, D187, D189, D203, D223, D243, D257, D292, D298, D301, D315, D397, D413, D432, D444, D445, D477, D484, D509, D570, D588, D610, D624 and D634 are deprotonated while D349 is protonated; (iii) glutamates: E95, E123, E144, E151, E182, E207, E251, E274, E276, E299, E398, E399, E404, E486, E499, E508, E545, E571, E582 and E640 are deprotonated, while E131 and E178 are protonated.

For the systems at pH = 4.5, the protonation states were as follows: (i) histidines: neutral Nδ for residues H82, H91, H171, H242, H378, neutral Nε for H539, and protonated for H30, H58, H113, H139, H185, H186, H226, H240, H262, H329, H356, H358, H416, H425, H449 and H572; (ii) aspartates: D35, D62, D69, D115, D119, D142, D145, D154, D184, D187, D189, D223, D243, D257, D292, D298, D301, D397, D413, D432, D444, D445, D477, D484, D509, D570, D588, D610, D624 and D634 are deprotonated, while D203, D315 and D349 are protonated; (iii) glutamates: E95, E123, E144, E151, E182, E207, E251, E274, E276, E299, E398, E399, E404, E486, E499, E508, E545, E571, E582 and E640 are deprotonated, while E131 and E178 are protonated.

Following this initial relaxation, the system was equilibrated and simulated under NPT conditions using the Martyna–Tobias–Klein (MTK) barostat and a Nosé–Hoover thermostat, with a relaxation time of 2 ps and 1 ps, respectively, maintaining the temperature at 310 K and the pressure at 1 atm. Long‐range electrostatic interactions were treated using the Gaussian split Ewald method, with a real‐space cutoff of 9.0 Å. Production MD simulations were carried out for a total of 1.6 μs across three independent replicas using Desmond Driver v2.3 (Schrödinger Suite 2024‐4) [42, 43]. A 2 fs integration time step was used, and system coordinates were saved every 100 ps. Before analysis, all trajectories were aligned using VMD to the backbone atoms (default C, Cα, N) of the first frame of the corresponding system and subsequently strided by a factor of 10, resulting in one frame every 1 ns. Based on post‐simulation analyses performed on the whole trajectory, i.e., the root mean square deviation (RMSD) of the protein's backbone, of the β‐sandwich alone (residues 398–542) and of the N372‐linked glycan, as well as the secondary structure analysis of the protein's backbone, the trajectories were divided into an initial 400 ns equilibration phase followed by a 1200 ns production phase. Therefore, subsequent analyses, namely, root mean square fluctuation (RMSF) of the glycan, clustering, principal component analysis (PCA), hydrogen bond analysis, Solvent‐Accessible Surface Area (SASA) and distance distributions, were calculated for the 400–1600 ns window.

The RMSD, RMSF and secondary structure analysis were calculated using MDAnalysis [45, 46], and hydrogen bond analysis was executed using the VMD version 2.0.0 alpha [47] ‘Hydrogen Bonds’ Module, and then plotted using matplotlib [48]. The RMSF values for the N372‐linked glycan were calculated by averaging the fluctuations of its nine constituent sugar residue. The clustering analysis was performed using an in‐house Python script with the scikit‐learn package, specifically the *k*‐means algorithm with *k* = 3, which was selected based on the best Silhouette scores for each system, namely WT, L490P and P533R (the three replicates were concatenated), separately (Table S2). PCA was performed after aligning all concatenated trajectories of all systems to the average Cα atoms of the WT structure, hereby generating a common PCA space. All heavy atoms of chain F, corresponding to the N372‐linked glycan, were included in the clustering and PCA analysis. SASA analysis was performed, via MDAnalysis, using a 5 Å cutoff from the centre of mass (com) of C53, E182 and E299. Distance distributions were calculated between the com of the glycan residue involved in each interaction and the C atom of the carboxylic group of the considered residue. The graphical representation was generated using matplotlib via Jupyter Notebook. The images were generated using PyMOL [49].

### 2.3 In Vitro Expression and Vector Preparation

IDUA untagged vector pCMV6‐XL4 containing human WT IDUA cDNA was purchased from Origene Technologies. Primers for c1469T>C p.L490P and for c.1598c>G p.P533R mutations were designed according to the manufacturer's instructions [50]. The mutations were inserted using the XL II Mutagenesis Kit from Agilent Technologies. The obtained mutant vectors (pCMV6‐XL4‐490P and pCMV6‐XL4‐533R) were expanded by the Miniprep procedure, Sanger‐sequenced to assess vector integrity and the absence of random mutations and finally stored at −80°.

### 2.4 COS7 Transfection and IDUA Biological Assay

Cos7 cells (Istituto Zooprofilattico Brescia) were plated in DMEM plus 10% FBS in T75 flasks and incubated at 37°C, 5% CO_2_. Cells were detached at confluence by trispin‐ EDTA treatment and plated at 1 × 10^4^/ml density in P60 dishes. After 24 h incubation, cells were transfected with 5 μg IDUA WT or mutant by lipofectamine 3000 transfection system (Thermofisher Scientific) [51]. 48 h post transfection, cells were lysed with whole‐cell extraction buffer (20 mM HEPES, 0.2 M NaCl, 0.5% Triton X‐100, 20% glycerol and 1 mM EDTA). The substrate of the IDUA, 4‐methylumbelliferyl α‐ʟ‐iduronide (4MU‐I, Toronto Research Chemicals, ON, Canada, #M334701), was prepared in sodium formate buffer (50 mM, pH 2.8) to a final concentration of 20 μg/20 μL. 20 μL aliquots of 4MU‐I were added to 20 μL of the cell extract. The mixture was incubated at 37°C for 1 h, and then 1000 μL glycine carbonate buffer (pH 10.5) was added to terminate the reaction. The intensity of emitted fluorescence resulting from the enzyme‐substrate reaction was measured with excitation at 365 nm and emission at 450 nm. A standard curve was prepared by serial dilution of 4MU (Sigma‐Aldrich, St. Louis, MO, USA, #M1381) from 0.45 to 30 nmol final concentration [52]. The enzyme activity was expressed as units per mg protein extract per hour.

## 3 Results

### 3.1 MD Simulations Suggest a Higher Conformational Rigidity of the N372‐Linked Glycan in Mutants

MD simulations were performed to compare the conformational landscape of the WT protein and the L490P and P533R mutants. These studies were conducted under two different pH conditions with three trajectory replicas generated for each system (namely R1, R2 and R3). Specifically, simulations were conducted at pH = 7.5 to reflect the ER microenvironment, where protein folding and maturation occur, and at pH = 4.5 to mimic the lysosomal microenvironment, where IDUA exerts its biological function, that is, the degradation of GAGs. Initial analyses were performed by calculating the RMSD of the protein backbone across all systems (Figure 2). From these graphs, we can infer that the systems reached equilibrium after approximately 400 ns. Notably, the backbone of both WT and mutants remains stable at both pH conditions.

**FIGURE 2:**
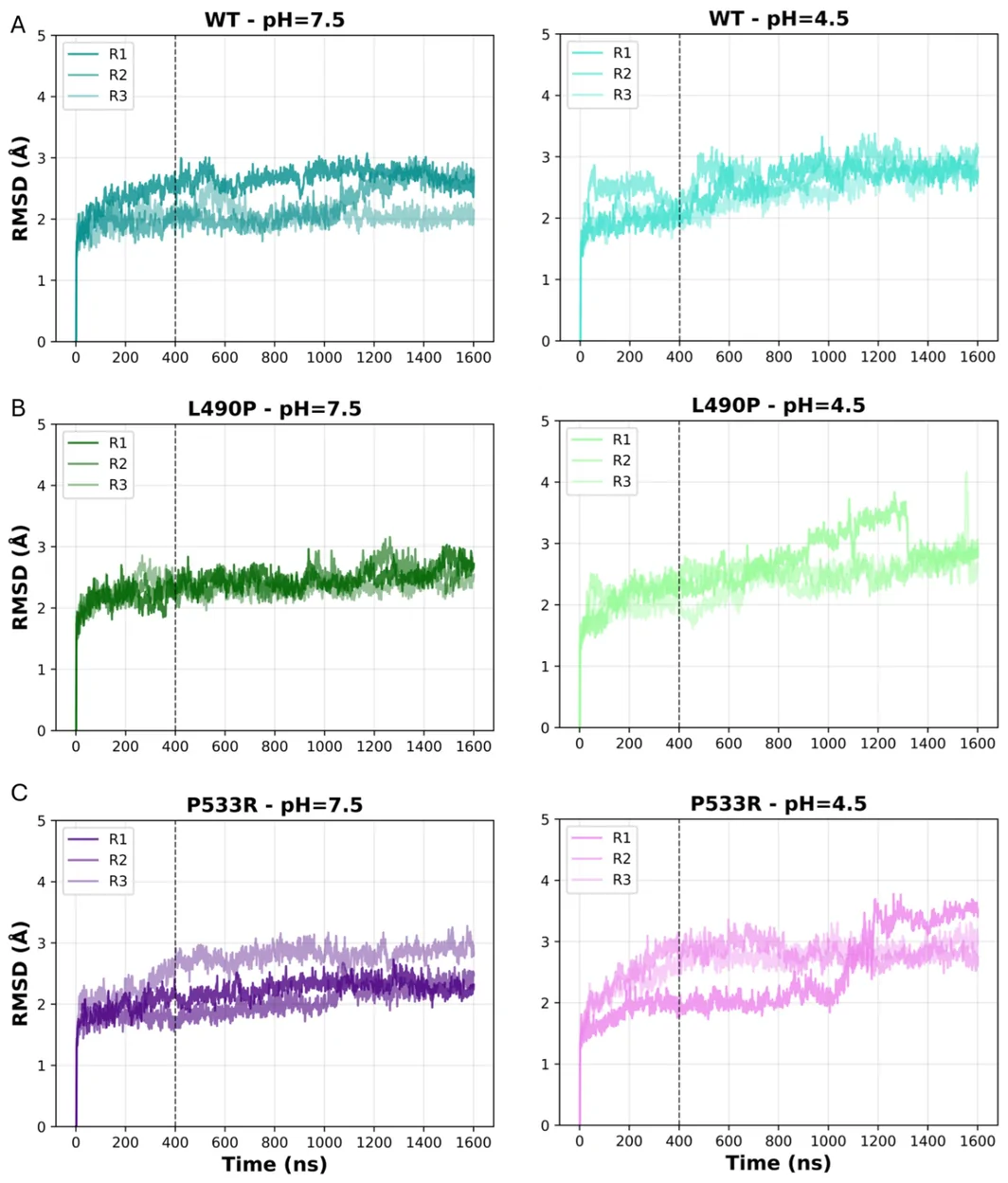
RMSD graphs of the IDUA backbone for each MD trajectory replica (namely R1, R2 and R3), subdivided into (A) WT pH 7.5 (teal) and pH 4.5 (cyan), on left and right, respectively, (B) mutant L490P pH 7.5 (forest green) and pH 4.5 (pale green), on left and right, respectively and (C) mutant P533R at pH 7.5 (purple) and pH 4.5 (pink), on left and right, respectively. Black dotted at 400 ns, indicating the cutoff for equilibration.

To assess whether these mutations destabilize the α‐helix and the β‐sandwich domain, as suggested by Bie et al. [4], we also plotted the RMSD values over time for the β‐sandwich (residues 398–542, where both mutated residues are located) across individual replicates. Remarkably, we did not detect any significant differences between the WT and the two mutants (Figure S1).

Additionally, from the secondary‐structure analysis, we observe that the groups represented (helix, strand, turn and coil) did not change over the simulation time (Figure S2). In particular, the percentage of secondary‐structure retention calculated for each system showed no substantial differences among the systems (Table 1). Overall, these results suggest that the mutations do not significantly affect the backbone secondary structure within the simulated timescale.

**TABLE 1:** Secondary structure composition occupancy and structural instability across simulated systems. For each system, the percentage of residues assigned to each secondary structure group (Helix, Strand, Turn and Coil) is averaged over the three concatenated replicates ± the standard deviation. Structural instability is defined as the percentage of residues whose dominant secondary structure group occupancy falls below the threshold of 85%, averaged across all three replicates.

System	WT		L490P		P533R	
pH	7.5	4.5	7.5	4.5	7.5	4.5
Helix	24.6 ± 0.9	24.5 ± 1.0	24.2 ± 0.9	24.3 ± 0.9	24.3 ± 0.9	24.6 ± 0.9
Strand	26.6 ± 1.2	25.6 ± 1.5	26.0 ± 1.3	26.0 ± 1.4	26.4 ± 1.1	25.8 ± 1.1
Turn	23.0 ± 1.1	23.5 ± 1.1	23.9 ± 1.2	23.7 ± 1.2	23.2 ± 1.1	23.3 ± 1.1
Coil*	0.6	0.6	0.6	0.6	0.6	0.6
Structural instability (< 85%)	42.4 ± 2.1	45.3 ± 1.4	44.5 ± 1.8	46.8 ± 2.7	44.2 ± 1.5	43.8 ± 2.4

* Standard deviation = 0.0.

Our data therefore suggest that the effect of the mutations may not involve global protein stability during folding under ER‐mimicking conditions at pH 7.5, but may instead become apparent at pH 4.5, corresponding to a lysosomal‐like environment. Due to the importance of the N372‐linked glycan, we focused our attention on its conformational changes throughout the production phase of our simulations, particularly under acidic pH conditions, where the mutation‐associated effects are more likely to emerge and potentially influence catalytic activity. Interestingly, analysis of the RMSD profiles of the N372‐linked glycan at pH 4.5 revealed higher RMSD values for the WT protein than for either mutant (Figure 3). In particular, the WT displays multiple glycan conformational states, whereas a more restricted conformational behaviour is observed in the mutants.

**FIGURE 3:**
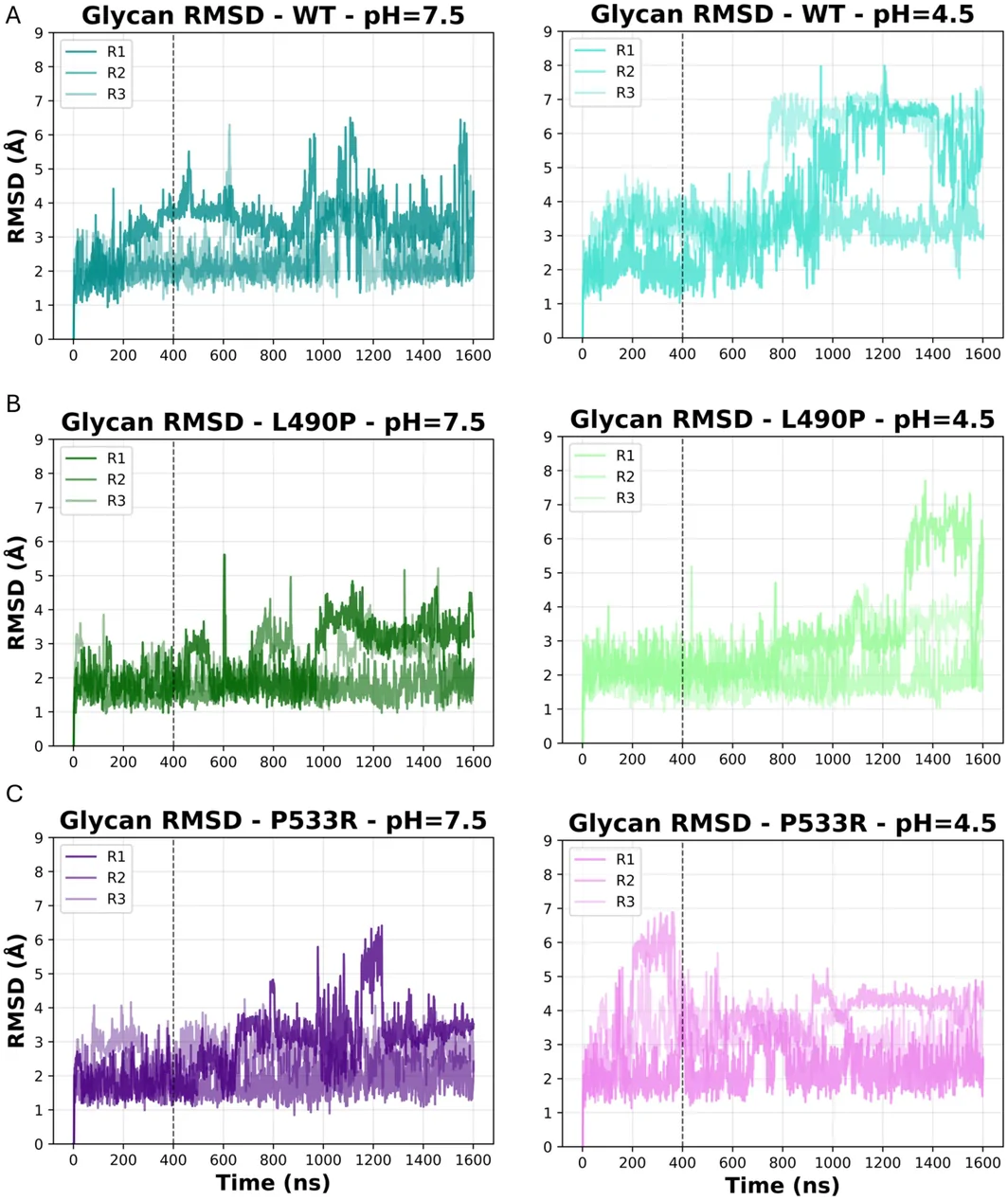
RMSD over time plots of the N372‐linked glycan for each replicate of the simulated trajectories at pH 4.5: (A) WT at pH = 7.5 (left in teal) and at pH = 4.5 (right in cyan), (B) L490P mutant at pH = 7.5 (left in forest green) and at pH = 4.5 (right in pale green) and (C) P533R mutant at pH = 7.5 (left in purple) and at pH = 4.5 (right in pink).

In this context, the literature highlights the importance of the N372‐linked glycan for the biological activity of IDUA. As reported by Maita et al. the N372‐linked glycan can directly interact with IdoA, and its occupancy positively correlates with IDUA enzymatic activity. Notably, these functional measurements were performed under acidic conditions, consistent with the lysosomal environment in which IDUA is active. These findings therefore support our observation that mutation‐induced structural alterations under lysosomal‐like conditions (pH = 4.5) may perturb the conformation and/or positioning of the N372‐linked glycan, ultimately compromising substrate recognition and catalytic efficiency [13].

Based on these observations, we further evaluated the RMSF of the glycan to assess whether the differences in conformational sampling were associated with variations in flexibility (Table S1). RMSF values, calculated over the 400–1600 ns time interval, did not display any significant changes between the WT and the mutants at pH 7.5. However, at pH 4.5, the glycan flexibility values are higher for the WT compared to the two mutants (WT: 6.438 ± 1.641 Å SEM; L490P: 3.456 ± 0.850 Å SEM; P533R: 3.531 ± 0.204 Å SEM). These results indicate that mutations at positions 490 and 533 may induce glycan rigidity, potentially contributing to the reduced IDUA activity.

### 3.2 PCA and Hydrogen Bond Analyses Reveal Distinct Conformational Spaces Occupied by the N372‐Linked Glycan via Specific Interactions with IDUA

To further characterize the conformational space sampled by the N372‐linked glycan, a PCA was performed on the WT, L490P and P533R systems at pH = 4.5, for the trajectories' production time (400–1600 ns). This approach reduces the dimensionality of the trajectory while retaining the dominant modes of motion. In the PCA plot (Figure 4A), Principal Component 1 (PC1), shown along the *x*‐axis, accounts for the greatest variance (69.5%), whereas PC2, along the *y*‐axis, explains an additional 10.6% of the total variance.

**FIGURE 4:**
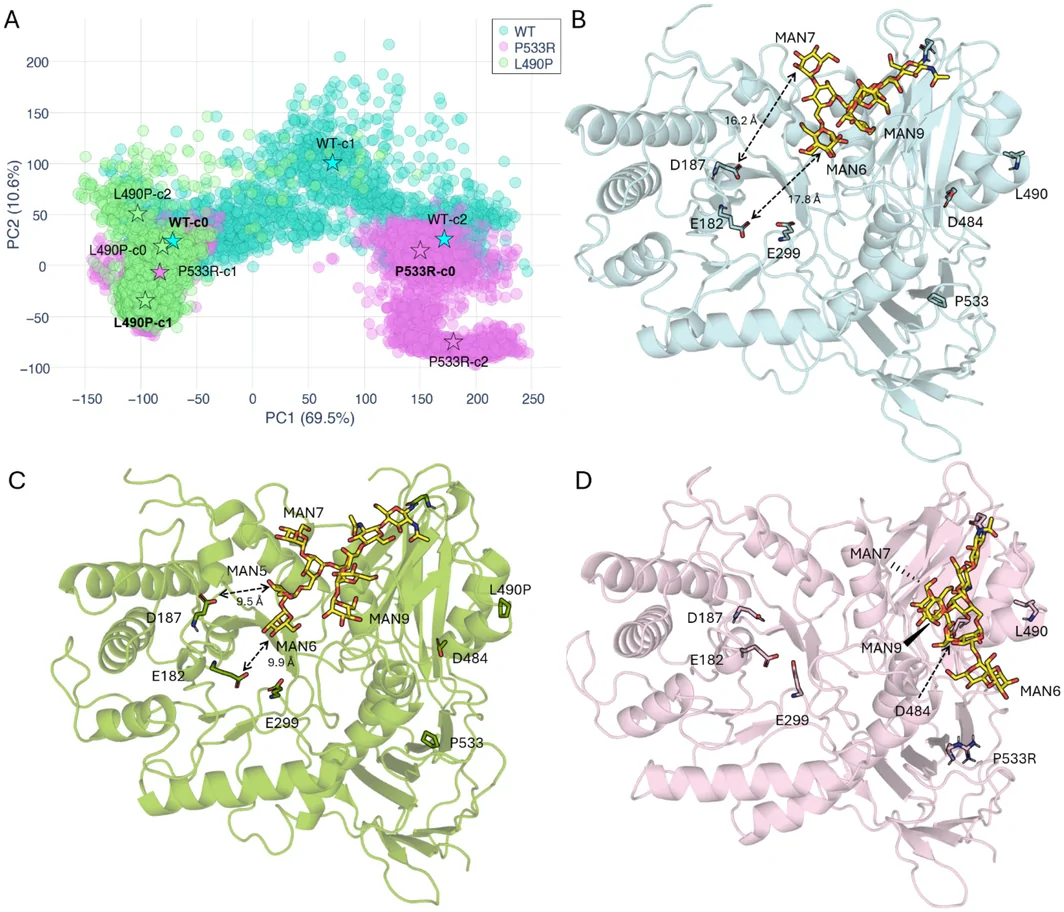
(A) PCA analysis of the conformation experienced by the N372 glycan in the three systems projected onto a shared PCA space (WT in cyan, L490P in green and P533R in pink). Stars indicate the positions of the representative frames of each cluster generated for the respective systems, as indicated by the corresponding labels; in bold the most populated clusters. Representative frames of the most populated cluster of each system, namely (B) WT‐c0 in cyan, (C) L490P‐c1 in green and (D) P533R‐c0 in pink; hashed wedged bond indicates the direction of MAN7 closer to the protein, while wedged bond of MAN9 towards the viewer. The N372‐linked glycan is represented in yellow sticks.

For the three different systems, namely WT, L490P and P533R at pH 4.5, a different distribution of the frames can be observed across their common PCA space. In particular, after cluster analysis of all three replicas for each system at pH 4.5, the returned representative frames of the most populated clusters (WT‐c0, L490P‐c1 and P533R‐c0; Figure 4A highlighted in bold) are distant from one another in their joint PCA space. This distribution indicates that both L490P and P533R mutants prefer conformations that are distinct from those observed in the WT (Figure 4B–D). We also checked whether the alternative conformers could affect the binding pocket area, specifically via SASA analysis, which confirms the previous observations; the WT is more accessible compared to the mutants (Figure S3).

Additionally, to rationalize the observed differences in conformational behaviour of the N372‐linked glycan and assess whether they are associated with the formation, stabilization, weakening or disruption of specific interactions, a hydrogen‐bond analysis was performed. Specifically, hydrogen bonds between the glycan and the protein with an occupancy greater than 10% across all three replicates of each system at pH = 4.5 are reported in Figure 5, as well as their distance distribution (Figure S4).

**FIGURE 5:**
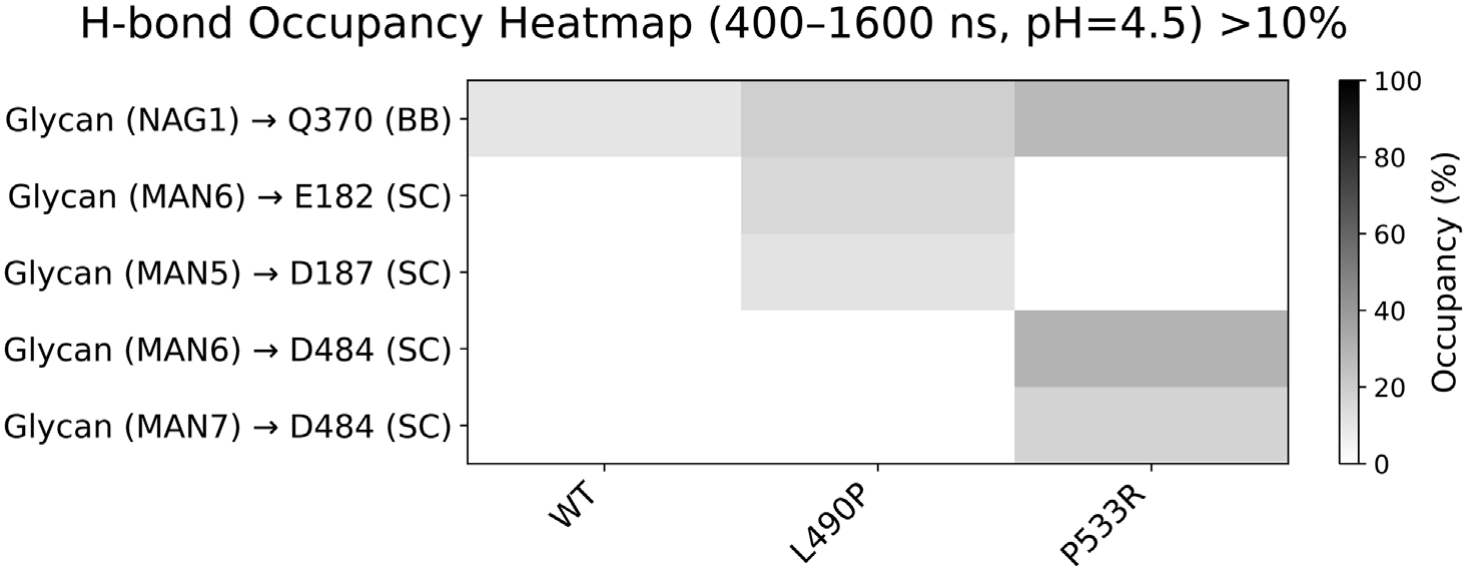
Occupancy in % of hydrogen bonds between the N372‐linked glycan and the protein for the range of 400–1600 ns of the trajectories at pH 4.5. Heatmap of all hydrogen bonds (Donor → Acceptor) for the four systems (WT and mutants L490P and P533R, at pH = 4.5) with an occupancy > 10% measured for the three replicates.

The hydrogen‐bond analysis returned specific residue‐glycan interactions with higher occupancy in the mutants with respect to the WT enzyme. In the L490P mutant, interactions between the glycan and residues E182 and D187 are observed (Figure S5A–C). These interactions display a non‐negligible occupancy (11.03% and 15.38%, respectively) in L490P and are practically absent in the WT form (0% and 0.11%, respectively). In particular, the impediment of the catalytic activity in the mutant L490P could potentially be attributed to the N372‐linked glycan's interaction with the residue E182, which is one of the key catalytic residues. The P533R mutant, on the other hand, exhibits a distinct interaction pattern, characterized by a hydrogen bond between the glycan and residue D484 with a significantly higher occupancy (29.70% for MAN6 and 17.42% for MAN7; Figure S5B–D). Remarkably, D484 is located in proximity to the mutated site (P533R; Figure 4D), suggesting that the enhanced interaction with the glycan may arise from local structural rearrangements induced by the mutation.

Finally, to confirm our in silico observations, we assayed the residual biological activity of both mutations in an in vitro COS7 expression cell system. According to this, the impaired enzymatic activity for both L490P and P533R may arise from common mutation‐dependent alterations in the conformational landscape of the N372‐linked glycan, known to be essential for IDUA activity. In other words, the tested mutations have comparable functional effects, in particular loss of enzymatic activity, compared to the WT. Indeed, their residual activity is 55% and 57% for L490P and P533R, respectively, compared to the WT (Figure S6).

## 4 Discussion

Overall, the MD simulations indicate that neither the L490P nor the P533R mutation induces substantial differences in backbone structural behaviour compared with the WT under either ER‐ or lysosome‐mimicking pH conditions. Instead, our analyses identify mutation‐associated differences in the conformational behaviour of the N372‐linked glycan at lysosomal pH, corresponding to the physiological environment in which IDUA catalyses GAG degradation. These results suggest that the impaired enzymatic activity may arise, at least in part, from mutation‐dependent alterations in the conformational landscape of this glycan, which has been reported to be important for IDUA activity [13].

At lysosomal pH, the WT exhibits higher N372‐linked glycan flexibility and samples a broader conformational space compared to both mutants, as indicated by RMSF analysis, clustering, and PCA analyses. Importantly, these differences are accompanied by changes in the glycan–protein interaction patterns, suggesting that altered local contacts may contribute to the observed conformational behaviour in each simulated system.

In L490P, the interaction of the glycan with the catalytic residue E182 provides a plausible mechanistic hypothesis for the impaired enzymatic activity, specifically the degradation of GAGs. In particular, the observed repositioning of the glycan towards the active‐site region could potentially influence substrate access or the local environment of the catalytic residues, although this proposed effect remains to be experimentally established. Analogously, in P533R, the glycan‐D484 interaction, absent in the WT, points to a distinct glycan conformation that may similarly contribute to impaired enzymatic activity, likely due to local structural rearrangements induced by the mutation. Notably, D484 is located close to the mutation site. Despite their different interaction signatures, both mutants converge on a common functional outcome—the restriction of glycan dynamics at lysosomal pH. Strikingly, this finding is consistent with preliminary in vitro results showing comparable residual activity for both mutants, supporting the hypothesis whereby their distinct structural perturbations converge on a similar functional outcome.

Taken together, these findings support a model in which disease‐associated mutations may impair human IDUA function not by disrupting the global protein fold, but by restricting the conformational freedom and orientation of the N372‐linked glycan. Hence, this glycan is not merely a passive post‐translational modification but may act as an active conformational element whose flexibility and orientation are possibly tightly coupled to enzymatic competence, thereby compromising enzymatic activity in the lysosomal environment; its perturbation by distal mutations may contribute to the residual but clinically significant enzymatic dysfunction characteristic of the Scheie phenotype. Therefore, our findings contribute to a more comprehensive understanding of the molecular mechanisms underlying MPS I pathogenesis and may provide a foundation for the rational design of therapeutic strategies aiming at modulating glycan–protein interactions or restoring productive glycan conformational dynamics, an aspect that remains largely underexplored.

## Author Contributions

**Maria Giovanna Eva Papadopoulos:** conceptualization, writing – original draft, methodology, formal analysis, data curation, visualization, investigation. **Giuseppe Bonapace:** writing – review and editing, investigation, methodology, formal analysis, validation. **Lorenzo Taglietti:** writing – review and editing. **Barbara La Ferla:** writing – review and editing, supervision. **Rosanna Rizzi:** funding acquisition, writing – review and editing. **Michele Pitaro:** writing – original draft. **Daniela Concolino:** writing – review and editing. **Domenico Alberga:** writing – review and editing, software, methodology, supervision. **Giuseppe Felice Mangiatordi:** conceptualization, writing – review and editing, writing – original draft, methodology, supervision, visualization, investigation.

## Funding

This work was funded by the European Union under the programme PRIN 2022 of the Italian Ministry of University and Research (MUR) (Project Prot. 2022S2M7E5, ‘Pharmacological Chaperon Therapy for Type I Mucopolysaccharidosis’, CUP F53D23002440006) and by the project ‘Potentiating the Italian Capacity for Structural Biology Services in Instruct Eric’, acronym ‘ITACA.SB’ (Project No. IR0000009, CUP B53C22001790006), funded by the European Union's NextGenerationEU under the MUR call 3264/2021 PNRR M4/C2/L3.1.1. We acknowledge ISCRA (Project No. HP10CZXBC2) for awarding this project access to the LEONARDO supercomputer, owned by the EuroHPC Joint Undertaking, hosted by CINECA (Italy).

## Ethics Statement

The authors have nothing to report.

## Consent

The authors have nothing to report.

## Conflicts of Interest

The authors declare no conflicts of interest.

## Supporting information


**Table S1**: List of simulated systems via molecular dynamics simulations.
**Table S2**: Clustering details and Silhouette scores of the three systems at pH = 4.5.
**Figure S1**: RMSD graphs of the β‐sandwich backbone region (residues 398–542) of IDUA, for each replicate of the simulated trajectories, subdivided into (A) WT pH 7.5 (left in teal) and pH 4.5 (right in cyan), mutant (B) L490P pH 7.5 (left in forest green) and pH 4.5 (right in pale green), and mutant (C) P533R at pH 7.5 (left in purple) and pH 4.5 (right in pink). Black dotted at 400 ns.
**Figure S2**: Secondary structure analyses of each system over time. The three replicates are concatenated and divided by a black dashed line. (A) WT at pH = 7.5, (B) WT at pH = 4.5, (C) L490P at pH = 7.5, (D) L490Pat pH = 4.5, (E) P533R at pH = 7.5 and (F) P533R at pH = 4.5.
**Figure S3**: SASA analysis of the binding pocket (COM residues C53, E182, E299 with a radius of 5 Å), over time for (A) WT, (B) L490P and (C) P533R mutants.
**Figure S4**: Graphical representation of the distance distributions of the observed hydrogen bonds: (A) between the WT and L490P, namely for the MAN6‐E182 (top) and MAN5‐D187 (bottom) hydrogen bonds, and (B) between the WT and P533R, namely for the MAN6‐D484 (top) and MAN7‐D484 (bottom). Kernel density estimate (KDE) of the distribution are also shown.
**Figure S5**: Structure of the frames with the shortest distance between the glycan and residues E182 and D187 for the L490P mutant (in green) and D484 for the P533R mutant (in pink) at pH = 4.5. Specifically, (A) frame 1037, replicate 2, distance = 4.456 Å between the COM of MAN6 and E182 (top), frame 567, replicate 2, distance = 4.195 Å between the COM of MAN5 and D187 (bottom) for the L490P mutant and (B) frame 570, replicate 3, distance = 3.992 Å between the COM of MAN6 and D484 (top), frame 1554, distance = 3.965 Å between the COM of MAN6 and D484 (bottom). For the sake of clarity, the point of view in B is rotated by ~90° on the *z*‐axis relative to A.
**Figure S6**: Residual activity of IDUA WT (red) and L490P, P533R mutants (green). WT = 254 ± 8 U/mg/h, L490P = 140 ± 2 U/mg/h (55% of WT) and P533R = 146 ± 5 U/mg/h (57% of WT). Statistical significance was assessed using an unpaired two‐tailed Student's *t*‐test, assuming normally distributed data and equal variances. Both mutants differed significantly from WT (*p* < 0.0001). Data are presented as mean ± SD (*n* = 4 independent experiments).

## Data Availability

The data that support the findings of this study are available from the corresponding author upon reasonable request.
