# Supplementary material for: Molecular Dynamics Reveals a Glycan Conformation Underlying IDUA Dysfunction in Attenuated MPS I
Source: J Cell Mol Med. 2026 Oct 11;30(19):e71396. doi: 10.1111/jcmm.71396 (PMC13654827; doi:10.1111/jcmm.71396)
Supplement: Supplementary file 1 [file JCMM-30-e71396-s001.docx]

**Molecular Dynamics Reveals a Glycan Conformation Underlying IDUA Dysfunction in Attenuated MPS I**

*Maria Giovanna Eva Papadopoulos,^a^ Giuseppe Bonapace,^b^ Lorenzo Taglietti,^c^ Barbara La Ferla,^d^ Rosanna Rizzi,^a^ Michele Pitaro,^e^ Daniela Concolino,^b^ Domenico Alberga^a^ and Giuseppe Felice Mangiatordi^a*^*

^a^ CNR – Institute of Crystallography, Via Amendola 122/o, 70126 Bari, Italy.

^b^ Department of Health Sciences, University Magna Graecia, Viale Europa, 80100 Catanzaro, Italy.

^c^ Department of Biotechnology and Biosciences, Milano-Bicocca University, Piazza della Scienza 1, 20126 Milan, Italy.

^d^ Department of Earth and Environmental Sciences, Milano-Bicocca University, Piazza della Scienza 1, 20126 Milan, Italy.

^e^ INBB (Istituto Nazionale Biostrutture e Biosistemi), Via dei Carpegna 19, 00165 Rome, Italy

*Correspondence: [giuseppefelice.mangiatordi@cnr.it](mailto:giuseppefelice.mangiatordi@cnr.it)

**Supporting Information**

[Table S1: List of simulated systems.	2](#_Toc241073208)

[Table S2: Clustering details.	4](#_Toc241073209)

[Figure S1: RMSD graphs.	5](#_Toc241073210)

[Figure S2: Secondary structure analyses.	6](#_Toc241073211)

[Figure S3: SASA analysis.	7](#_Toc241073212)

[Figure S4: Distance distributions	8](#_Toc241073213)

[Figure S5: Frames with the shortest distances.	9](#_Toc241073214)

[Figure S6: Residual activity of IDUA.	10](#_Toc241073215)

# **Table S1:** List of simulated systems via molecular dynamics simulations.

| **System** | **pH** | **backbone RMSD (Å)^a^** | | | **glycan RMSF (Å)^b^** | | |
| --- | --- | --- | --- | --- | --- | --- | --- |
|  |  | Rep1 | Rep2 | Rep3 | Rep1 | Rep2 | Rep3 |
| WT | 7.5 | 2.595 ± 0.252 | 2.184 ± 0.341 | 2.036 ± 0.187 | 5.856 ± 2.078 | 2.782 ± 1.367 | 2.245 ± 1.020 |
| P533R | 7.5 | 2.187 ± 0.224 | 2.014 ± 0.259 | 2.664 ± 0.326 | 6.225 ± 2.026 | 1.913 ± 0.824 | 2.234 ± 0.780 |
| L490P | 7.5 | 2.411 ± 0.244 | 2.420 ± 0.245 | 2.036 ± 0.187 | 3.663 ± 1.448 | 2.312 ± 0.884 | 2.103 ± 1.087 |
| WT | 4.5 | 2.356 ± 0.358 | 2.468 ± 0.394 | 2.323 ± 0.172 | 7.652 ± 2.213 | 8.471 ± 2.395 | 3.189 ± 1.173 |
| P533R | 4.5 | 2.415 ± 0.665 | 2.722 ± 0.311 | 2.682 ± 0.327 | 3.177 ± 1.094 | 3.883 ± 1.248 | 3.534 ± 1.651 |
| L490P | 4.5 | 2.647 ± 0.501 | 2.473 ± 0.250 | 2.309 ± 0.360 | 5.107 ± 2.081 | 2.281 ± 0.932 | 2.979 ± 1.274 |

^a^Mean RSMD values ± standard deviation calculated for the total length of the trajectories (0-1600 ns).

^b^Mean RMSF values ± standard deviation returned by the residues of the N372-linked glycan in the production time (400-1600 ns).

# **Table S2:** Clustering details and Silhouette scores of the three systems at pH=4.5.

| **System** | **Population** | | | **Production**  **(concatenated)** | **Silhouette scores** | | |
| --- | --- | --- | --- | --- | --- | --- | --- |
|  | **c0** | **c1** | **c2** |  | **k=2** | **k=3** | **k=4** |
| WT | 2058 | 763 | 785 | 1.2 μs × 3 | 0.544 | 0.522 | 0.454 |
| P533R | 1187 | 1202 | 1217 | 1.2 μs × 3 | 0.664 | 0.670 | 0.623 |
| L490P | 750 | 2552 | 307 | 1.2 μs × 3 | 0.444 | 0.421 | 0.393 |

**Figure S1:** RMSD graphs of the β-sandwich backbone region (residues 398-542) of IDUA, for each replicate of the simulated trajectories, subdivided into A) WT pH 7.5 (left in teal) and pH 4.5 (right in cyan), mutant B) L490P pH 7.5 (left in forest green) and pH 4.5 (right in pale green), and mutant C) P533R at pH 7.5 (left in purple) and pH 4.5 (right in pink). Black dotted at 400 ns.

**Figure S2:** Secondary structure analyses of each system over time. The three replicates are concatenated and divided by a black dashed line. A) WT at pH=7.5, B) WT at pH=4.5, C) L490P at pH=7.5, D) L490Pat pH=4.5, E) P533R at pH=7.5, and F) P533R at pH=4.5.

**Figure S3:** SASA analysis of the binding pocket (COM residues C53, E182, E299 with a radius of 5 Å), over time for A) WT, B) L490P and C) P533R mutants.

**Figure S4:** Graphical representation of the distance distributions of the observed hydrogen bonds: A) between the WT and L490P, namely for the MAN6-E182 (top) and MAN5-D187 (bottom) hydrogen bonds, and B) between the WT and P533R, namely for the MAN6-D484 (top) and MAN7-D484 (bottom). Kernel density estimate (KDE) of the distribution are also shown.

**Figure S5:** Structure of the frames with the shortest distance between the glycan and residues E182 and D187 for the L490P mutant (in green) and D484 for the P533R mutant (in pink) at pH=4.5. Specifically, A) frame 1037, replicate 2, distance = 4.456 Å between the COM of MAN6 and E182 (top), frame 567, replicate 2, distance = 4.195 Å between the COM of MAN5 and D187 (bottom) for the L490P mutant, and B) frame 570, replicate 3, distance = 3.992 Å between the COM of MAN6 and D484 (top), frame 1554, distance = 3.965 Å between the COM of MAN6 and D484 (bottom). For the sake of clarity, the point of view in B is rotated by ~90° on the z-axis relative to A.

**D187**

**E182**

**MAN6**

**MAN5**

**D484**

**MAN6**

**MAN7**

**D484**

A	B

C	D

# **Figure S6:** Residual activity of IDUA WT (red) and L490P, P533R mutants (green). WT = 254 ± 8 U/mg/h, L490P = 140 ± 2 U/mg/h (55% of WT) and P533R = 146 ± 5 U/mg/h (57% of WT). Statistical significance was assessed using an unpaired two-tailed Student's t-test, assuming normally distributed data and equal variances. Both mutants differed significantly from WT (p < 0.0001). Data are presented as mean ± SD (n = 4 independent experiments).
